# Higher dental caries rates and increased cardiovascular disease risk

**DOI:** 10.3389/froh.2025.1571148

**Published:** 2025-10-13

**Authors:** Catherine Roberts, Dylan J. Baxter, Dobrawa Napierala, Mariana Bezamat

**Affiliations:** Department of Oral and Craniofacial Sciences, School of Dental Medicine, University of Pittsburgh, Pittsburgh, PA, United States

**Keywords:** dental caries, NHANES, cardiovascular disease, DMFT (decayed missing and filled teeth), DMFS index

## Abstract

**Introduction:**

Epidemiological studies on the association between dental caries and cardiovascular disease accounting for shared risk factors are inconclusive. This cross-sectional study aimed to determine independent associations between quantitative indices of dental caries including the decayed missing and filled surfaces/ teeth (DMFS/DMFT) and cardiovascular disease in populations from two large dental and medical datasets.

**Methods:**

We used data from the Dental Registry and DNA Repository (DRDR) comprising 2,247 individuals and the National Health and Nutrition Examination Survey (NHANES) that included 3,202 participants. We hypothesized that there would be a significant association between dental caries with cardiovascular disease, when accounting for traditional risk factors. Using R software and STATA, we conducted multiple regression models accounting for risk factors while controlling for multiple testing to determine associations.

**Results:**

The DRDR participants were more likely to report a history of cardiovascular disease (23.97% vs. 18.06% in the NHANES) and, in general, had higher overall DMFT scores (19.58 vs. 14.78 in the NHANES). After accounting for age, sex, smoking, and ethnicity, DMFS was associated with cardiovascular disease in the DRDR population (*p* < 0.006), and DMFT was significantly associated with cardiovascular disease in the NHANES dataset (*p* < 0.0001) accounting for age, sex, smoking, income, periodontal bone loss, and periodontal treatment.

**Conclusions:**

Our results show that participants with higher occurrence of dental caries are more likely to have a history of cardiovascular disease independently of traditional risk factors and confounders.

**Clinical relevance:**

The DMFT and DMFS indices could be explored for inclusion in cardiovascular disease prediction tools and future clinical use if causality is established.

## 1 Introduction

Dental caries is a multifactorial disease characterized by destruction of hard tissues including dental enamel and dentin (1). In more severe cases, inflammation of dental pulp can occur by penetration of microorganisms in the root canal through deeper cavities (2). It has been suggested that these microorganisms can enter the blood stream and cause other health issues, including systemic inflammation (3). The association of microorganism infection as well as increased inflammatory responses suggest a link between dental and cardiovascular disease. *Streptococcus mutans*, for instance, is a major etiological agent of dental caries (4) and has been found in heart valves and atheromatous plaques (5), suggested as an indirect risk factor for the association with infective endocarditis (6) and involvement with accelerated atherosclerotic plaque growth in mice (7).

There is an abundancy of published literature on the association between periodontitis and cardiovascular disease (8–12), but less epidemiologic research has been done on dental caries and this association remains inconclusive (12–14). A cohort study showed an association between dental caries and risk of coronary heart disease (CHD) among middle-aged men and women in the Republic of Korea. Their findings demonstrate that higher outpatient visits for dental caries was significantly associated with an increase in CHD risk but the number of decayed and treated teeth was not included in the analysis (15). The authors speculate that severe dental caries may induce chronic inflammatory response in cardiovascular cells and accentuate cardiometabolic risk factors that contribute to heart disease (15). Other cardiovascular diseases have been associated with severity of dental caries including risk of stroke (14) and endocarditis (6).

Most prior studies used small sample sizes, limited populations, lacked the inclusion of the number of decayed and treated teeth and did not control for important risk factors like smoking. Thus, this present cross-sectional study assessed these associations in populations from two different large dental/medical databases with detailed and quantitative dental phenotypes while controlling for risk factors: The University of Pittsburgh, School of Dental Medicine, Dental Registry and DNA Repository (DRDR) and the National Health and Nutrition Examination Survey (NHANES). The DRDR database has about 7,000 dental school clinic participants that consented to be part of the registry and includes demographic data, dental pathologies at the time of their examination, and self-reported medical history (16). After applying the exclusion criteria, 2,247 participants data were included. The NHANES provided by the Center of Disease Control and Prevention (CDC) (17) survey data included 3,202 individuals across the US, with detailed dental information, self-reported health history, and demographic information.

In summary, epidemiologic studies allow for investigation into disease relationships on large scales by compiling data from multiple resources about several aspects of participants lives. An epidemiological analysis with both survey and clinical data was performed to verify the connection between cardiovascular disease and oral health while accounting for traditional risk factors associated with both diseases. We hypothesize that there will be a statistically significant association between quantitative indices of dental caries including the decayed missing and filled surfaces/teeth due to caries (DMFS/DMFT) and a history of cardiovascular disease, when accounting for traditional risk factors like age, sex, smoking, ethnicity, periodontal disease, and income.

## 2 Methods

### 2.1 Datasets and data preparation

#### 2.1.1 Dental registry and DNA repository (DRDR)

At the University of Pittsburgh, School of Dental Medicine, we have a database of about 7,000 dental school clinic participants that consented to be part of the registry. The DRDR project has the approval of the University of Pittsburgh Institutional Review Board (IRB # STUDY19050020) and all participants signed a written informed consent. The registry includes deidentified demographic data, dental pathologies at the time of their examination, and self-reported ethnicity and medical history (18). The recruitment for the included participant data occurred between 2006 and 2022. For the purposes of this analysis, patients under 45 years of age were excluded since cardiovascular disease usually affects older adults (19). Therefore, only data from 2,247 DRDR participants older than 45 years were included.

Patients in the DRDR were considered to have a history of cardiovascular disease if they answered, ‘Yes’ to the following questions: ‘Heart Surgery?’, ‘Artificial Heart Valves?’, ‘Irregular Heartbeat?’, ‘Congenital Heart Lesions?’, ‘Heart Murmur?’, or ‘Mitral Valve Prolapse?’ Those who answered ‘No’ to all these questions also had the Comments and Summary of Medical Risk Assessment categories checked for the key words ‘clot’, ’stroke’, ‘cardiac’, ‘coronary’, ‘carotid’, ‘carditis’, or ‘heart’, with those only mentioning ‘heart burn’ being excluded from our experimental group. With a database query, a list of patients who met any of the former or latter conditions was created. Then, excel was used to cross-check these lists and create a column of binary (yes/no) cardiovascular disease data. Subjects were classified according to their smoking habits as ever using tobacco or being a current tobacco user or not. Non-smoking was used as the baseline for analysis. Detailed DRDR participant demographics is provided in Table 1.

**Table 1:** Participant demographics.

DRDR cohort (*n* = 2,247)		
Variable	*n*/mean	%/range
Age in years (mean, range)	60.3	(45–97)
Sex (*n*, %)		
Female	1,155	51.4%
Male	1,092	48.6%
Oral disease indices		
DMFT (mean, range)	19.8	(0–28)
DMFS (mean, range)	76.2	(0–128)
Smoking (*n*, %)		
Smoker	597	26.6%
Non-smoker	1,641	73%
Missing	9	0.4%
Cardiovascular disease (*n*, %)		
No CVD	1,707	76%
CVD	540	24%
NHANES Cohort (*n* = 3,202)		
Variable		
Age in years (mean, range)	62.3	(45–80)
Sex (n, %)		
Female	1,777	55.5%
Male	1,425	44.5%
Oral disease indices		
DMFT (mean, range)	14.8	(0–28)
Periodontitis (*n*, %)	644	20.1%
Smoking (*n*, %)		
Non-smoker	2,082	65%
Some days	221	6.9%
Every day	899	28.1%
Cardiovascular disease (*n*, %)		
No CVD	2,601	81.2%
CVD	601	18.8%

#### 2.1.2 National health and nutrition examination survey (NHANES)

We explored the data provided by the CDC through the NHANES, a publicly accessible database. Each year, the NHANES survey is conducted on approximately 5,000 individuals across the US who consented to be part of the survey. The data from this survey provides detailed dental information, health history, and demographic information. This data is open for public use as provided by the CDC National Center for Health Statistics and ethical approval is not required. This survey included an oversampling of minority and older adult populations (20). We used NHANES data from 2015 to early of 2020, which includes two surveys (21, 22).

The NHANES data needed to be cross-linked between surveys using each participant’s Respondent Sequence Number (SEQN) to connect each person's age, found in Demographics Data, to their corresponding Medical Conditions questionnaire and Oral Health-Dentition report.

Python was used to select columns of variables from each dataset and combine them into one CSV file containing data from all three questionnaires from 2015 to 2020. We selected: 1) sequence number, gender, age, and income from the demographic data; 2) sequence number and answers to the heart disease-related questions from the medical questionnaire; and 3) sequence number and coronal caries codes for each tooth from the dental examination dataset. Participants missing dental examination information or health questionnaire data or those with invalid data were excluded from the study. We then combined datasets into one file based on sequence number. A total of 6,878 participants were identified, however, participants missing smoking data were also removed from analysis and a total of 3,202 individuals were included in the logistic regression.

Patients in the NHANES dataset were considered to have a history of cardiovascular disease if they answered, ‘Yes’ to the following questions: ‘Ever told had congestive heart failure?’, ‘Ever told you had coronary heart disease?’, ‘Ever told you had angina/angina pectoris?’, ‘Ever told you had heart attack?’, or ‘Ever told you had a stroke?’ Python was used to loop through these rows, checking for a positive response, and create a column of binary (yes/no) heart disease data.

The DRDR reports dental caries through Decayed, Missing, or Filled Teeth (DMFT) and Decayed, Missing, or Filled Surfaces (DMFS) scores, while the NHANES dataset reports on the condition of each tooth. We calculated a DMFT score from this NHANES individualized data by tallying all coronal caries condition codes not recorded as Code S: Sound Permanent Tooth. These other codes include pathologies under the umbrella of decayed, missing, or filled teeth. The most common among these is Code F: Permanent tooth with a restored surface condition. Excel was used to tally the DMFT for each patient so that the data is comparable to the DMFT scores provided in the DRDR.

The NHANES included smoking habits information from the questionnaire. Smoking was divided into several categories. For the purposes of this analysis, smoking habits were divided into three categories; non-smoker, smokes some days a week, and smokes every day. Non-smoking was used as the baseline for analysis. Detailed NHANES participant demographics is provided in Table 1.

Both databases contain age and smoking habits information, but income data was only provided by the NHANES. This was presented as ratio of income over the calculated poverty line for the time and location of the participant. Participants with income below the poverty line would have an income ratio less than 1, while those above the poverty line have an income ratio greater than 1.

### 2.2 Software and statistical analysis

#### 2.2.1 Relationship of risk factors with cardiovascular disease and dental caries

Using R, we ran an analysis of variance of cardiovascular disease with risk factors for both NHANES and DRDR datasets. Additional analysis of variance of DMFT and DMFS with risk factors for NHANES and DRDR were conducted. With the NHANES dataset, we ran an analysis of variance of cardiovascular disease with income ratio and a Pearson's correlation test of DMFT with income ratio. Since two phenotypes (DMFT and DMFS) were tested, Bonferroni correction was used to correct for multiple comparisons and *p*-values below 0.025 (0.05/2) were considered significant.

#### 2.2.2 Dental caries association with cardiovascular disease

**Figure 1:**
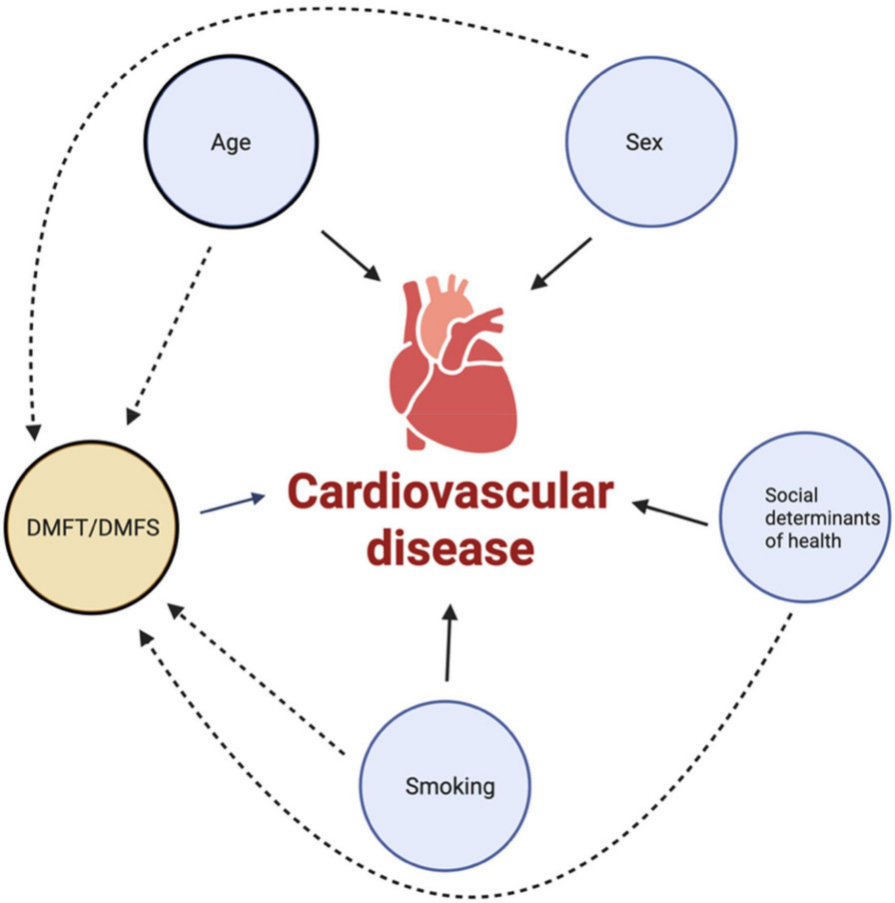
Illustration of classic and proposed cardiovascular disease risk factors included in the analyses. Created in BioRender. Bezamat Chappel, M. (2025) https://BioRender.com/6yoe3a2.

To analyze the relationship between dental caries and cardiovascular disease, a logistic regression model was used in Stata SE. The model was created using cardiovascular disease as the outcome and age, sex, income ratio, DMFT, periodontal bone loss, gingivitis treatment, and smoking status as covariates in the NHANES population. In the DRDR, the model was created using cardiovascular disease as the outcome and DMFS, age, sex, smoking, and ethnicity as covariates. Figure 1 illustrates the proposed and classic cardiovascular disease risk factors included in the analysis. Bonferroni correction was also conducted and only *p*-values below 0.025 were considered significant. The model accounted for each covariate individually as well as overall in the model. The fitness of the model was also calculated using the goodness of fit function in Stata SE as well as tests to verify the association of covariates with each other.

## 3 Results

### 3.1 Relationship of risk factors with cardiovascular disease and dental caries

The analysis of variance of cardiovascular disease with age was statistically significant in both the NHANES (*p* < 2 × 10^−16^) and DRDR (*p* = 3.87 × 10^−16^) datasets after multiple testing correction was performed. DMFT and age were also significantly correlated in both NHANES (*p* < 2 × 10^−16^) and DRDR (*p* = 2.44 × 10^−14^). This shows that even though patients under 45 were excluded from both datasets before analysis, the effect of age on disease in older adults remains in all cases.

**Figure 2:**
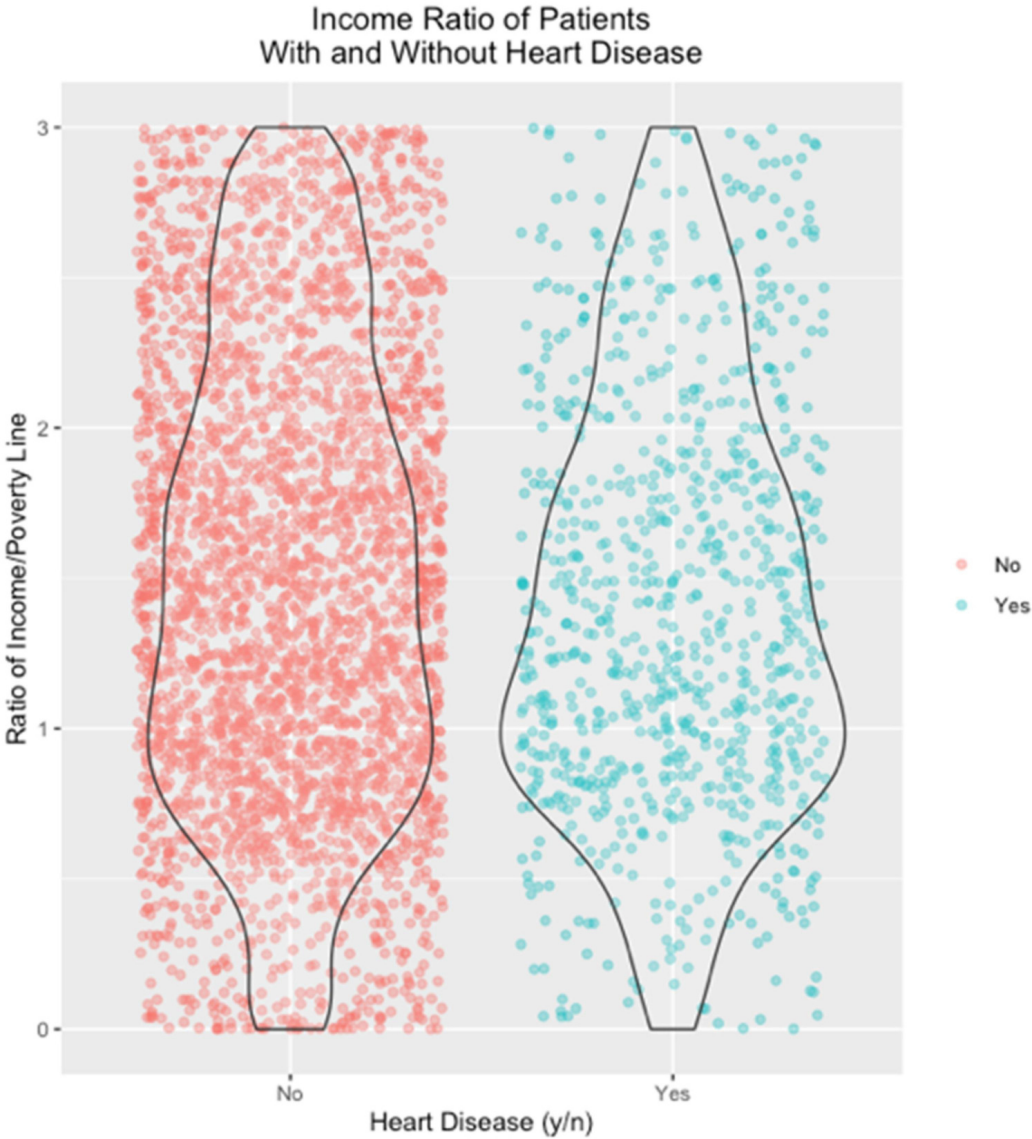
Participants with cardiovascular disease have high frequency of income near the poverty line in NHANES 2015–2020 surveys.

**Figure 3:**
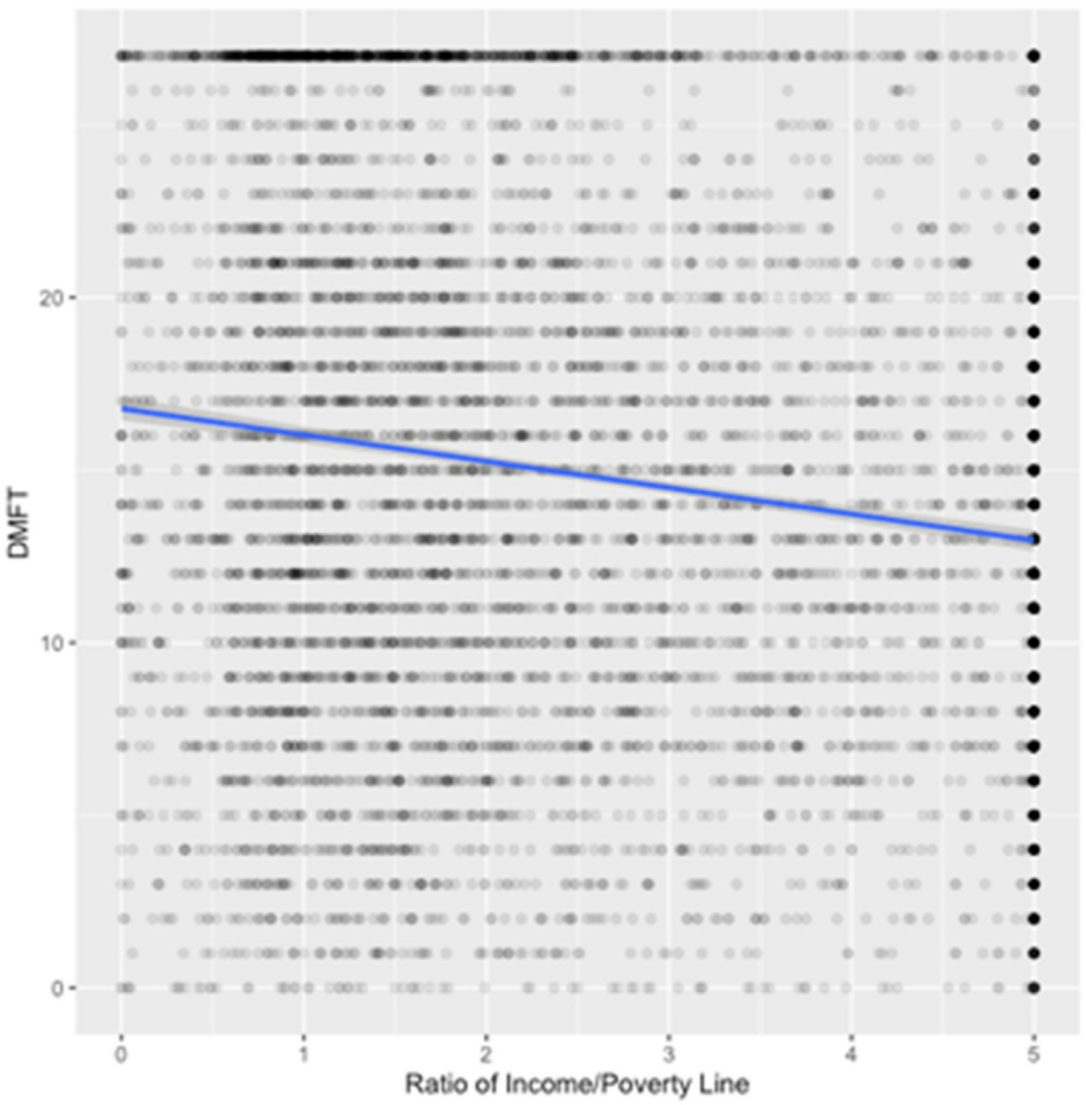
Decayed, missing, or filled teeth (DMFT) vs. income ratio in NHANES 2015–2020 survey. As income ratio increases, DMFT decreases (oral health improves).

**Figure 4:**
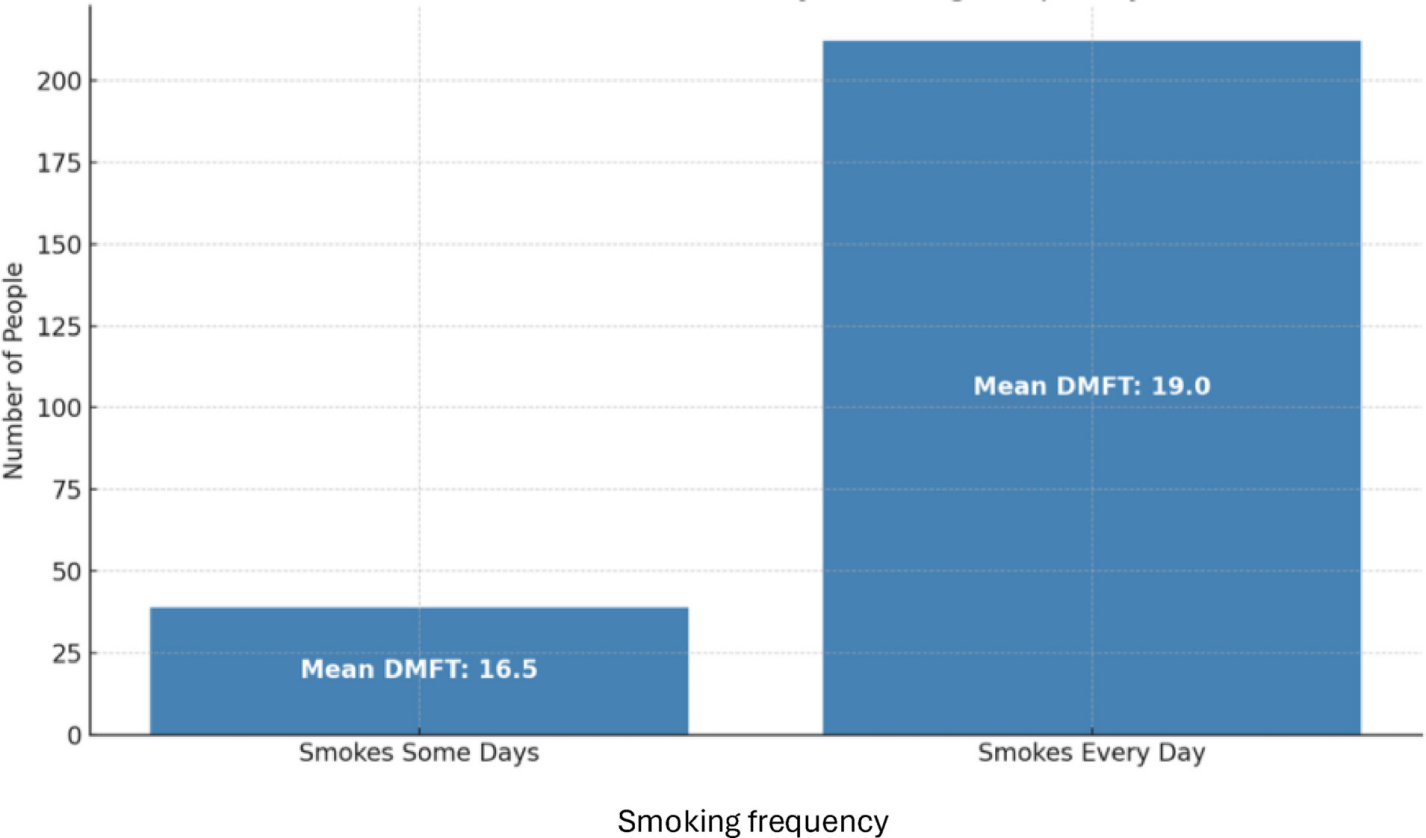
Participants with a history of cardiovascular disease have higher DMFT if they reported to be everyday smokers than if they only smoke some days in the NHANES population.

In the NHANES dataset, the analysis of variance of cardiovascular disease with income ratio showed a significant association (*p* < 2 × 10^−16^, Figure 2), and a Pearson's correlation test of DMFT with income ratio (*p* < 2 × 10^−16^) was also significantly associated (Figure 3). Both cardiovascular disease and DMFT were very strongly correlated with income ratio. The violin/jitter plot (Figure 2) and a scatterplot with a best fit line (Figure 3) illustrate these relationships. Figure 2 shows that participants with cardiovascular disease are more likely to live near the poverty line, while those without are more evenly distributed among higher income brackets. Additionally, in Figure 3, as income ratio increases a participant's number of decayed, missing, or filled teeth decreases, indicating better oral health. Smoking was significantly associated with cardiovascular disease in both the DRDR (*p* = 0.01) and NHANES (*p* < 2 × 10^−16^). Smoking was also associated with DMFT in the DRDR (*p* = 0.001) and in the NHANES dataset (*p* < 2 × 10^−16^). To further explore smoking data in relation to DMFT and cardiovascular disease, we analyzed the DMFT of CVD participants who smoke “some days” and smoke “every day” (Figure 4). Participants with a history of cardiovascular disease have higher DMFT if they reported to be everyday smokers (DMFT means = 19) than if they only smoke some days (DMFT mean = 16.5) in the NHANES population (Figure 4).

### 3.2 Dental caries association with cardiovascular disease

Model fitness was assessed using a goodness-of-fit test (*p* = 0.74), indicating an acceptable fit to the data. In this test, the null hypothesis is that the model fits the data well, a *p*-value above 0.05 indicates insufficient evidence to reject the null hypothesis, supporting the adequacy of the model fit (23, 24). The logistic regression between cardiovascular disease and DMFT in the DRDR dataset detected borderline but no significant association when accounting for risk factors and multiple testing (*p* = 0.044, Table 2). This *p*-value is just outside of a standard 95% confidence interval. However, the DRDR database also includes DMFS scores for each participant, which were significantly associated with cardiovascular disease after accounting for all other covariates (*p* = 0.006, Table 3). This value is significant within a 99% confidence interval.

**Table 2:** Logistic regression results on DRDR population.

Covariates	Odds ratio	Std. error	z	*P*>|z|	Lower 95% C.I.	Upper 95% C.I.
Age	1.04103	0.0055737	7.51	0	1.030162	1.052011
DMFT	1.013086	0.0065399	2.01	0.044	1.000349	1.025986
Sex (Female)	0.8724736	0.0882612	−1.35	0.177	0.715555	1.063804
Smoking	1.133496	0.1398227	1.02	0.31	0.8900609	1.443511
Ethnicity	1.106856	0.128882	0.87	0.383	0.8810032	1.390608
Constant	0.0178691	0.0066171	−10.87	0	0.0086476	0.0369242

Cardiovascular disease is the outcome and DMFT is included as a covariate along with age, sex, smoking, and ethnicity.

**Table 3:** Logistic regression results on DRDR population.

Covariates	Odds ratio	Std. error	z	*P*>|z|	Lower 95% C.I.	Upper 95% C.I.
Age	1.038951	0.0056711	7	0	1.027895	1.050126
DMFS	1.003978	0.0014516	2.75	0.006	1.001137	1.006828
Sex (Female)	0.8728459	0.0883821	−1.34	0.179	0.7157265	1.064457
Smoking	1.175136	0.1469562	1.29	0.197	0.9196897	1.501533
Ethnicity	1.108174	0.1291209	0.88	0.378	0.8819191	1.392475
Constant	0.0186873	0.0067767	−10.97	0	0.0091806	0.0380384

Cardiovascular disease is the outcome and DMFS is included as a covariate along with age, sex, smoking, and ethnicity.

**Figure 5:**
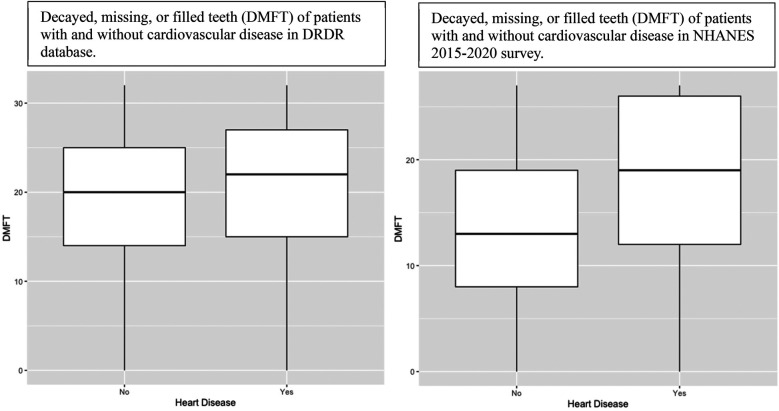
Participants with a history of cardiovascular disease have higher DMFT than those without. This difference is greater in the NHANES population.

The association between cardiovascular disease and DMFT, accounting for all risk factors, in the NHANES dataset was also significant (*p* < 0.0005, Table 4). The significance of the relationship between DMFT and cardiovascular disease was much greater in the NHANES dataset than in the DRDR population. R was used to create a box and whisker plot of DMFT of participants with and without cardiovascular disease for each database. Figure 5 shows that the average DMFT value is higher among participants with cardiovascular disease in both datasets, but that this difference in means is greater in the NHANES dataset.

**Table 4:** Logistic regression results on NHANES population.

Covariates	Odds ratio	Std. error	z	*P*>|z|	Lower 95% C.I.	Upper 95% C.I.
Sex (Female)	0.7144839	0.0658632	−3.65	0	0.5963843	0.8559702
Age	1.052795	0.0053913	10.05	0	1.042281	1.063415
Income ratio	0.8635316	0.026032	−4.87	0	0.8139879	0.9160909
DMFT	1.031841	0.0066762	4.84	0	1.018839	1.045009
Smoking_1	0.9353264	0.1808368	−0.35	0.729	0.6403113	1.366266
Smoking_2	1.211267	0.1289954	1.8	0.072	0.9830829	1.492415
Gingivitis treatment	1.045143	0.0545962	0.85	0.398	0.9434318	1.157819
Periodontal bone loss	0.9899718	0.0466305	−0.21	0.831	0.9026695	1.085718
Constant	0.0095493	0.0032504	−13.66	0	0.0049005	0.0186083

Cardiovascular disease is the outcome and DMFT is included as a covariate along with age, income ratio, gingivitis treatment, and periodontal bone loss.

Smoking_1 = Smokes some days, Smoking_2 = Smokes every day.

In the total population of each dataset, the mean value of DMFT is 14.78 (or rounded up to 15 decayed, missing, or filled teeth) in the NHANES participants, a lower value than the mean DMFT of the DRDR participants: 19.62 (or rounded up to 20 decayed, missing, or filled teeth, Table 1). Excluding the high count of answers at the maximum in both datasets, the widest part of the NHANES plot is around 10–15 DMFT while the DRDR contains few values this low, widening from 15 to 25 (Figure 5).

DRDR participants also were more likely to report a history of cardiovascular disease. In total, 24% of participants in the DRDR database indicated a history of cardiovascular disease, while 18.8% reported cardiovascular disease in their NHANES survey questionnaire (Table 1**)**. Despite being on average older, with a mean age of 62.3, the NHANES participants have better reported overall oral and cardiovascular health than the DRDR population, with a mean age of 60.3 (Table 1).

In summary, the logistic regression model shows that cardiovascular disease is associated with DMFT when accounting for traditional risk factors (listed covariates) with *p* < 0.0001 in the NHANES population and *p*-values were also significant for sex, age, and income ratio (Table 4). Odds ratio results were also above 1 for age, DMFT, and daily smoking (Table 4).

## 4 Discussion

In the analysis of variance, age, smoking, and income are strongly associated with dental caries and cardiovascular disease, even after applying Bonferroni correction for multiple testing, as previously reported (25–27). In the logistic regression, the association between higher DMFT scores and cardiovascular disease in NHANES remained significant after accounting for all risk factors. Higher DMFS scores were also associated with cardiovascular disease in the DRDR when controlling for age, sex, smoking, and ethnicity but this relationship was stronger in the NHANES dataset. This is particularly notable as the NHANES data includes individuals not presently in need of clinical dental care. As shown, this dataset contains individuals with better reported oral (lower DMFT) and cardiovascular health. The DRDR participants have more indicators of poor health despite being slightly younger on average. Additional research is needed to understand the cause of this contrast, but a plausible explanation is that the DRDR is a registry of individuals who actively seek dental care in a clinical setting, where the data was collected. Further, some of these differences may be caused by differences in demographics (geographic location, literacy, income, etc.) between the two datasets.

Our results are in line with published literature. A prior study demonstrated an association between severity of dental caries and risk of stroke in a large Asian population (14). However, smoking habits were not controlled for in their analysis which is a major risk factor for both oral and cardiovascular diseases. In our study, we had available data on smoking habits which was a strength, and we demonstrated that dental caries is associated with cardiovascular disease independently from traditional risk factors, including smoking. In line with previous studies, we found that in the NHANES population, participants with a history of cardiovascular disease have higher mean DMFT if they reported to be everyday smokers than if they only smoke some days (19 vs. 16.5, respectively). Another recent cohort study showed an association between dental caries and risk of coronary heart disease (CHD) among middle-aged men and women in the Republic of Korea. Their findings demonstrate that higher outpatient visits for dental caries was significantly associated with an increase in CHD risk but they did not take the number of decayed and treated teeth into account (15). Recently, additional research using NHANES data found a moderate association between dental caries and hypertension (28). Similarly to our present study, the authors underscore the importance of oral conditions serving as indicators of broader general health issues. The authors discussed that their limitations included not considering participant age as a confounder (28), which we were able to address in this present study. Additional prior limitation that we accounted for was the use of quantitative dental caries measures (DMFT and DMFS) rather than binary classification of caries presence (yes/no). This is important because as we discussed in prior research (29), having one tooth affected by caries (DMFT = 1) involves different factors from having 10 teeth affected by the disease (DMFT = 10). Using DMFT and DMFS indexes account for severity of disease.

Our data supports that more quantitative markers of oral health such as the DMFT and DMFS might be a powerful way of identifying risk if a causal relationship is determined with further research. Participants with more severe dental caries scores are more likely than the average participant to have a history of cardiovascular disease. Though the Odds Ratio can be considered small (1% per tooth affected), when considering a full mouth, even small increases per tooth could lead to a meaningful impact in clinical outcome.

As suggested before, a plausible mechanism involved in this association includes genetics that may play a role in increasing susceptibility to both dental and cardiovascular disease (30). As such, since the DRDR is also a DNA repository, we hope to identify single nucleotide variants (SNVs) related to higher disease susceptibility in the future. However, these associations may also be a result of disparities in access to health and oral care that increase susceptibility to both oral and systemic health conditions (31). Machine learning models to predict risk of cardiovascular disease through dental analysis may also be considered as the field evolves and these associations are established, and potential causality is confirmed with additional research. Diseases such as dental caries and periodontal disease, easily identifiable by a dentist, may be indicative of underlying, systemic conditions. Thus, quantitative markers of oral health combined with all other traditional risk factors such as income and access to care could help impact cardiovascular disease outcomes (32). Although it is difficult to predict whether clinicians will accept and routinely use these oral health markers for cardiovascular disease risk assessment, their adoptions will likely depend on how readily healthcare systems are willing to integrate such metrics. Recently, mobile applications for caries risk assessment have been developed (33) and could eventually be expanded and tested for cardiovascular diseases. This is a cost-effective preventive strategy that could have impactful outcomes especially for high-risk populations.

Limitations of the study include the survey nature of some dental and medical data. Survey data can at times be unreliable because patients do not always recall each condition they have or are sometimes not honest about all conditions or disease state severity. The DRDR also lacks the specifics of income data that NHANES provides, but it does have more specific dental data. Additional limitations were the lack of inclusion of other confounders that may be involved with cardiovascular disease etiology such as BMI, sugar and salt intake, physical activity, and oral hygiene habits. The cross-sectional design of this study does not account for temporality of outcome and causality. Lack of inclusion of former smokers also likely had an impact on the findings. Tobacco use has lifelong physiological implications in the body, particularly for oral and cardiovascular health, however; former smoking status is a widely varied category that could potentially skew meaningful results. Someone who only smoked for a short time then quit would not see as great of a permanent physiological impact compared to someone who smoked for most of their life and has only quit for a short time. Furthermore, cardiovascular disease is a wide category of diseases and the combination of different definitions on cardiovascular disease and dependence on questionnaire responses, although positively impacted the power of analyses, were limitations of the study. Thus, these results should be taken cautiously and need validation with different populations.

Although there were limitations of our study, there were also strengths in the results generated; our sample size was powerful to detect the associations with the inclusion of covariates. In addition, both datasets generated results that are in line with literature reports of associations between dental and systemic health, and more importantly, we demonstrate for the first time that quantitative indices of dental caries (DMFT and DMFS) are associated with cardiovascular disease independently of traditional risk factors.

For future validation of these results, an integration between dental and medical databases are recommended, the creation of an integrated electronic health record system would be impactful in allowing for an expansion of research focusing on the bidirectionality of oral-systemic health relationships (34). In conclusion, our results show that participants with higher dental caries scores including DMFT and DMFS are more likely to have a history of cardiovascular disease independently of risk factors for these conditions.

## Data Availability

The NHANES is a publicly available dataset and the University of Pittsburgh School of Dental Medicine DRDR dataset analyzed is available from the corresponding author on reasonable request.

## References

[1] Larsen T Fiehn NE. Dental biofilm infections—an update. APMIS. (2017) 125(4):376–84. 10.1111/apm.12688 28407420

[2] Pitts NB Zero DT Marsh PD Ekstrand K Weintraub JA Ramos-Gomez F Dental caries. Nat Rev Dis Primers. (2017) 3:17030. 10.1038/nrdp.2017.30 28540937

[3] Li X Kolltveit KM Tronstad L Olsen I. Systemic diseases caused by oral infection. Clin Microbiol Rev. (2000) 13(4):547–58. 10.1128/CMR.13.4.547 11023956 PMC88948

[4] Loesche WJ. Role of Streptococcus mutans in human dental decay. Microbiol Rev. (1986) 50(4):353–80. 10.1128/mr.50.4.353-380.1986 3540569 PMC373078

[5] Soto-Barreras U Olvera-Rubio JO Loyola-Rodriguez JP Reyes-Macias JF Martinez-Martinez RE Patino-Marin N Peripheral arterial disease associated with caries and periodontal disease. J Periodontol. (2013) 84(4):486–94. 10.1902/jop.2012.120051 22680302

[6] Nomura R Matayoshi S Otsugu M Kitamura T Teramoto N Nakano K. Contribution of severe dental caries induced by Streptococcus mutans to the pathogenicity of infective endocarditis. Infect Immun. (2020) 88(7). 10.1128/IAI.00897-19 32312765 PMC7309618

[7] Kesavalu L Lucas AR Verma RK Liu L Dai E Sampson E Increased atherogenesis during Streptococcus mutans infection in ApoE-null mice. J Dent Res. (2012) 91(3):255–60. 10.1177/0022034511435101 22262633 PMC3275337

[8] Sanz M Del Castillo AM Jepsen S Gonzalez-Juanatey JR D'Aiuto F Bouchard P Periodontitis and cardiovascular diseases. Consensus report. Glob Heart. (2020) 15(1):1. 10.5334/gh.400 32489774 PMC7218770

[9] Lamprecht R Rimmele DL Schnabel RB Heydecke G Seedorf U Walther C Cross-sectional analysis of the association of periodontitis with carotid intima media thickness and atherosclerotic plaque in the Hamburg city health study. J Periodontal Res. (2022) 57(4):824–34. 10.1111/jre.13021 35675038

[10] Dietrich T Sharma P Walter C Weston P Beck J. The epidemiological evidence behind the association between periodontitis and incident atherosclerotic cardiovascular disease. J Periodontol. (2013) 84(4 Suppl):S70–84. 10.1902/jop.2013.134008 23631585

[11] Alhadainy HA Keefe T Abdel-Karim AH Abdulrab S Halboub E. Association between dental diseases and history of stroke in the United States. Clin Exp Dent Res. (2021) 7(5):845–51. 10.1002/cre2.416 33797859 PMC8543477

[12] Bezamat M Saeed A McKennan C Duan J Zhou R Baxter DJ Oral disease and atherosclerosis may be associated with overlapping metabolic pathways. JDR Clin Trans Res. (2024) 13:23800844241280383. 10.1177/23800844241280383 PMC12359100 39385367

[13] Donders HCM LM IJ Soffner M van 't Hof AWJ Loos BG de Lange J. Elevated coronary artery calcium scores are associated with tooth loss. PLoS One. (2020) 15(12):e0243232. 10.1371/journal.pone.0243232 33326424 PMC7743922

[14] Ono Y Chou YC Chien WC Sun CA. Association between severity of dental caries and the risk of stroke. Oral Dis. (2023) 30(5):3413–21. 10.1111/odi.14756. 37864387

[15] Kim K Choi S Chang J Kim SM Kim SJ Kim RJ-Y Severity of dental caries and risk of coronary heart disease in middle-aged men and women: a population-based cohort study of Korean adults, 2002–2013. Sci Rep. (2019) 9(1):10491. 10.1038/s41598-019-47029-3 31324851 PMC6642137

[16] Gianos E Jackson EA Tejpal A Aspry K O'Keefe J Aggarwal M Oral health and atherosclerotic cardiovascular disease: a review. Am J Prev Cardiol. (2021) 7:100179. 10.1016/j.ajpc.2021.100179 34611631 PMC8387275

[17] Liu C Foti K Grams ME Shin JI Selvi E. Trends in self-reported prediabetes and metformin use in the USA: NHANES 2005–2014. J Gen Intern Med. (2020) 35(1):95–101. 10.1007/s11606-019-05398-5 31637644 PMC6957593

[18] Bezamat M Harrison B Zhou Y Glickman KM Telles V Guirguis C Phenome-wide scan finds potential orofacial risk markers for cancer. Sci Rep. (2020) 10(1):4869. 10.1038/s41598-020-61654-3 32184411 PMC7078198

[19] Yazdanyar A Newman AB. The burden of cardiovascular disease in the elderly: morbidity, mortality, and costs. Clin Geriatr Med. (2009) 25(4):563–77. vii. 10.1016/j.cger.2009.07.007 19944261 PMC2797320

[20] Chen S Stubblefield A Stoner JA. Oversampling of minority populations through dual-frame surveys. J Surv Stat Methodol. (2021) 9(3):626–49. 10.1093/jssam/smz054 34322557 PMC8308969

[21] NHANES databases. Centers for Disease Control and Prevention (CDC). 2015–2016. Available online at: https://wwwn.cdc.gov/nchs/nhanes/continuousnhanes/default.aspx?Cycle=2017-2020

[22] NHANES databases. Centers for Disease Control and Prevention (CDC). 2017–March 2020. Available online at: https://wwwn.cdc.gov/nchs/nhanes/continuousnhanes/default.aspx?Cycle=2017-2020

[23] Hosmer DW Lemeshow S. Applied Logistic Regression. Wiley Series in Probability and Statistics Texts and References Section. 2nd ed New York: Wiley (2000). p. 373. xii.

[24] LaValley MP. Logistic regression. Circulation. (2008) 117(18):2395–9. 10.1161/CIRCULATIONAHA.106.682658 18458181

[25] Griffin SO Jones JA Brunson D Griffin PM Bailey WD. Burden of oral disease among older adults and implications for public health priorities. Am J Public Health. (2012) 102(3):411–8. 10.2105/AJPH.2011.300362 22390504 PMC3487659

[26] Eke PI Dye BA Wei L Thornton-Evans GO Genco RJ. Prevalence of periodontitis in adults in the United States: 2009 and 2010. J Dent Res. (2012) 91(10):914–20. 10.1177/0022034512457373 22935673

[27] Joshy G Arora M Korda RJ Chalmers J Banks E. Is poor oral health a risk marker for incident cardiovascular disease hospitalisation and all-cause mortality? Findings from 172 630 participants from the prospective 45 and up study. BMJ Open. (2016) 6(8):e012386. 10.1136/bmjopen-2016-012386 27577588 PMC5013478

[28] Natarajan P Madanian S Marshall S. Investigating the link between oral health conditions and systemic diseases: a cross-sectional analysis. Sci Rep. (2025) 15(1):10476. 10.1038/s41598-025-92523-6 40140465 PMC11947117

[29] Bezamat M Deeley K Khaliq S Letra A Scariot R Silva RM Are mTOR and endoplasmic reticulum stress pathway genes associated with oral and bone diseases? Caries Res. (2019) 53(3):235–41. 10.1159/000492675 30205378 PMC6411456

[30] Loos BG Van Dyke TE. The role of inflammation and genetics in periodontal disease. Periodontol 2000. (2020) 83(1):26–39. 10.1111/prd.12297 32385877 PMC7319430

[31] Northridge ME Kumar A Kaur R. Disparities in access to oral health care. Annu Rev Public Health. (2020) 41:513–35. 10.1146/annurev-publhealth-040119-094318 31900100 PMC7125002

[32] Bezamat M. An updated review on the link between oral infections and atherosclerotic cardiovascular disease with focus on phenomics. Front Physiol. (2022) 13:1101398. 10.3389/fphys.2022.1101398 36589419 PMC9794572

[33] S CS S A Madan Kumar PD. Comparison of dental caries risk assessment using CaRisk- a simple mobile based application and WHO deft, DMFT scores: a cross sectional study. J Dent (Shiraz). (2024) 25(2):138–46. 10.30476/dentjods.2023.98075.2051 38962073 PMC11217057

[34] Acharya A Shimpi N Mahnke A Mathias R Ye Z. Medical care providers’ perspectives on dental information needs in electronic health records. J Am Dent Assoc. (2017) 148(5):328–37. 10.1016/j.adaj.2017.01.026 28284418

